# When may an adult woman with cognitive impairment still have capacity to consent to an abortion?

**DOI:** 10.5694/mja2.52719

**Published:** 2025-07-02

**Authors:** Julia P Duffy, Sam Boyle, Casey M Haining

**Affiliations:** ^1^ University of Technology Brisbane QLD; ^2^ University of Melbourne Melbourne VIC

**Keywords:** Medicolegal, Reproductive rights, Abortion, induced, Obstetrics, Pregnancy, Informed consent

All Australian jurisdictions have now decriminalised access to abortion care.^1^, ^2^, ^3^, ^4^, ^5^, ^6^, ^7^, ^8^ This has been a critical step towards ensuring women's reproductive freedom and has only been achieved nationally with final legislation commencing in Western Australia in March 2024.^9^ A person can access abortion care provided all legislative and procedural requirements are met and they give informed consent. Consent requires capacity to make the decision and that it is given voluntarily, without coercion or undue influence. As with other medical procedures, medical practitioners who perform abortions without lawful consent may be subject to civil or criminal actions for assault or disciplinary proceedings.^10^

Complexities arise when people with cognitive impairment — whether associated with intellectual disability, acquired brain injury or mental illness — request an abortion. In this article, we examine the limited number of recent cases in Australia on legal capacity to consent to an abortion for adult women, distilling key factors to consider. No cases were found relating to people seeking abortion care identifying other than as women, and the language we use reflects this.

## Legal capacity to consent

When it is uncertain whether a woman has capacity to consent to an abortion or termination of pregnancy (we use the terms interchangeably), the usual legal pathway is to apply under guardianship or health care decision‐making legislation to an administrative tribunal for a determination on capacity and request the tribunal's consent to perform the procedure. This is the process to be followed whether or not the woman is already subject to a guardianship order (Box 1). This article is limited in focus on the important issue of capacity, and not on the procedure for obtaining consent for a termination from the tribunal.

Box 1 **Guardianship and health care decision‐making legislation on capacity and consent to abortion**

**Table:** 

State/territory	Legislation	Resources
Australian Capital Territory	*Guardianship and Management of Property Act 1991* ss 69‐70, “prescribed medical procedure”	https://www.ptg.act.gov.au/ https://www.acat.act.gov.au/
New South Wales	*Guardianship Act 1987* part 5 *Guardianship Regulation 2016* r 9, “special treatment”	https://www.nsw.gov.au/departments‐and‐agencies/trustee‐guardian https://ncat.nsw.gov.au/
Northern Territory	*Health Care Decision‐Making Act 2023* ss 30, 41, “restricted health care”	https://pgt.nt.gov.au/ https://ntcat.nt.gov.au/
Queensland	*Guardianship and Administration Act 2000* ss 68‐71, “special health care”	https://www.publicguardian.qld.gov.au/ https://www.qcat.qld.gov.au/
South Australia	*Guardianship and Administration Act 1993* s 61, “prescribed treatment”	https://www.opa.sa.gov.au/ https://www.sacat.sa.gov.au/
Tasmania	*Guardianship and Administration Act 1995* part 6, div 2, “special treatment”	https://www.publicguardian.tas.gov.au/ https://www.tascat.tas.gov.au/
Victoria	*Guardianship and Administration Act 2019* part 6, “special medical procedure”	https://www.publicadvocate.vic.gov.au/ https://www.vcat.vic.gov.au/
Western Australia	*Guardianship and Administration Act 1990* Part 9D, div 2, subd 3	https://www.wa.gov.au/organisation/department‐of‐justice/office‐of‐the‐public‐advocate https://sat.justice.wa.gov.au/

Capacity is always presumed, so incapacity must be proven.^10^ To assess capacity, legislation mostly adopts a functional test, which focuses on the process of decision making, that is, whether an adult can sufficiently understand the decision they are making and communicate their decision in some way.^11^ This test is intended to avoid determining capacity based on whether the decision is considered by others to be wise, reasonable or ethical, although commentators note that sometimes reasonableness considerations can impinge.^12^

In WA, the guardianship legislation expressly provides that to have capacity, a person must be able to make “reasonable judgements”.^13^ However, in case *C*, the relevant tribunal imported the functional test to complement the test of reasonableness (Box 2).^14^

Box 2 ***C* [2024] WASAT 50 (*‘C’*)**

**Table:** 

**Age:** 34 years.
**Gestation period at time of hearing:** 11 weeks.
**Procedure:** Surgical abortion.
**Clinical circumstances:** The woman “*AB*” had a longstanding history of schizophrenia and polysubstance use disorder. She had been in hospital for treatment of her schizophrenia for four months. Her symptoms had greatly improved, and she was no longer an involuntary patient, but still in hospital voluntarily and adhering to her medication regime.
**Social circumstances:** Normally lived at home, with her mother's support.
**Capacity assessment for other matters:** Previously found to have impaired capacity for decisions on health care, service provision and finances. Guardian for those matters and administrators currently appointed.
**Evidence from:** *AB*, mother, treating doctors, psychiatrists, obstetrician, social workers and guardian.
**Nature of evidence:** *AB* wanted an abortion because she did not know who the father was and did not want to have a baby without adequate supports. She had two abortions previously. The psychiatrist said she had not been influenced by any psychotic delusions in her desire for an abortion. The obstetrician said she canvassed all options with *AB* who could repeat back the risks of the procedure.
**Tribunal's reasoning for finding of capacity:** *AB* conveyed her wishes very clearly. Finding of incapacity for other personal matters and finances did not necessarily mean that *AB* lacked capacity for the abortion decision. The tribunal must consider the reasoning process undertaken. A person does not have to demonstrate a “sophisticated medical knowledge” to be able to make the decision. It is sufficient that a person is “capable of understanding the main elements of the procedure, and its risks and consequences, rather than the technical or exact details of the treatment or its effect” [paragraph 45 of the judgement]. Her reasons suggested a “rational approach” to making her choice [paragraph 54 of the judgement].

Capacity tests are also time‐ and decision‐specific.^10^ If a woman will soon regain capacity, then medical practitioners must wait until that time so that she can make the abortion decision herself. However, delaying decisions may be problematic, given that, as the pregnancy progresses, there may be additional legislative (and sometimes procedural) requirements and increased clinical complexity.^15^

Finally, guardianship legislation in Queensland, Victoria, Tasmania, the Northern Territory and the Australian Capital Territory (Box 1) either mandates or encourages providing supports for people to exercise legal capacity and make their own decisions.^16^

## The cases

To explore how cognitive impairments affect a woman's capacity to consent to termination of pregnancy, we searched for relevant cases using the Australian Legal Information Institute and administrative tribunal databases. We identified only five decisions after the decriminalisation of access to abortion care (Box 3). This may not be the total number of decisions made because tribunals publish decisions discretionally, are not obliged to provide reasons for decision unless requested, and do not publicise how many abortion‐related decisions they make.

For the remainder of this section, we distil key ethical and legal considerations stemming from the cases. *LKZ* was the only case where a finding of incapacity was made (Box 3). *LKZ*'s cognitive impairment was caused by a sudden onset medical condition, and the evidence was uncontested and uncontroversial. We therefore focus only on the remaining four cases (Box 2, Box 4, Box 5 and Box 6).

Box 3 **Cases on capacity to consent to an abortion**

**Table:** 

Case	Tribunal	Requested abortion?	Outcome
*BSE* [2020] QCAT 494 (https://www.queenslandjudgments.com.au/caselaw/qcat/2020/494)	Queensland Civil and Administrative Tribunal	Yes	Capacity
*GKB* [2020] NSWCATGD 99 (https://www.austlii.edu.au/cgi‐bin/viewdoc/au/cases/nsw/NSWCATGD/2020/99.html?context=1;query=GKB;mask_path=au/cases/nsw/NSWCATGD)	New South Wales Civil and Administrative Tribunal	Yes	Capacity
*LKZ* [2023] QCAT 315 (https://www.austlii.edu.au/cgi‐bin/viewdoc/au/cases/qld/QCAT/2023/315.html?context=1;query=LKZ;mask_path=au/cases/qld/QCAT)	Queensland Civil and Administrative Tribunal	Unable to indicate	Impaired capacity. Authorised termination
*C* [2024] WASAT 50 (Application relating to woman, ‘AB’) (https://www.austlii.edu.au/cgi‐bin/viewdoc/au/cases/wa/WASAT/2024/50.html)	Western Australia State Administrative Tribunal	Yes	Capacity
*FXB* [2024] NSWCATGD 14 (https://www.austlii.edu.au/cgi‐bin/viewdoc/au/cases/nsw/NSWCATGD/2024/14.html?context=1;query=FXB;mask_path=au/cases/nsw/NSWCATGD)	New South Wales Civil and Administrative Tribunal	Yes	Capacity

Box 4 ***BSE* [2020] QCAT 494 (‘*BSE*’)**

**Table:** 

**Age:** 19 years.
**Gestation period at time of hearing:** 15 weeks.
**Procedure:** Surgical abortion.
**Clinical circumstances:** Mental illness and severe and regular self‐harming.
**Social circumstances:** National Disability Insurance Scheme (NDIS) participant with daily support. In short stay mental health unit.
**Capacity determinations for other matters:** Impaired capacity to make decisions on accommodation, services (including for the NDIS) and finances. Guardian for those matters currently appointed. The Public Guardian had also been making decisions on general health care matters as *BSE*'s Statutory Health Attorney. Guardian for legal matters revoked because *BSE*'s legal issues in the Magistrate's Court had been finalised.
**Evidence from:** *BSE*, gynaecologist, psychiatrist, guardian.
**Nature of evidence:** The psychiatrist found capacity to consent. Capacity fluctuated depending on situational and emotional stressors, but when seen alone and not distressed, *BSE* had the ability to weigh up the advantages and disadvantages of her personal decisions, including the decision on the abortion. *BSE* could understand her options, including the risks and benefits of proceeding with the pregnancy as well as the longer term issues of caring for a baby.
**Tribunal's reasoning for finding of capacity:** Abortion is a one‐off decision even though very serious. In other areas where impaired capacity had been found (accommodation and services), there were requirements for ongoing decision making with significant consequences. Although *BSE* may have impaired capacity for some personal decisions, she did have capacity to consent to the abortion, understanding the nature and effect of the decision. She very clearly told the tribunal her views and wishes.

Box 5 ***GKB* [2020] NSWCATGD 99 (‘*GKB*’)**

**Table:** 

**Age:** 18 years.
**Gestation period at time of hearing:** 10 weeks.
**Procedure:** Surgical abortion.
**Clinical circumstances:** Fragile X syndrome.
**Social circumstances:** Other family members also had fragile X syndrome diagnoses. National Disability Insurance Scheme (NDIS) participant. Interpreters used although not essential.
**Capacity assessments for other matters:** Nil. Earlier psychologist's report advising low level of intellectual functioning.
**Evidence from:** *GKB*, social worker, NDIS support coordinator, obstetrician, general practitioner.
**Nature of evidence:** *GKB* repeated continually that she did not want to be pregnant, was too young to have a baby, and wanted to get back to school. She explained that she understood that she would be sleeping during the operation, that there would be bleeding and that the baby would be taken out. She did not respond when asked if she understood the risks of the operation, without it being clear if this was due to a lack of understanding or mere fatigue experienced in the hearing.
**Tribunal's reasoning for finding of capacity:** Given the nature and weight of the evidence, her functioning was still such that she could understand the facts relevant to the decision and make the decision.

Box 6 ***FXB* [2024] NSWCATGD 14 (‘*FXB*’)**

**Table:** 

**Age:** 30 years.
**Gestation period at time of hearing:** 19 weeks.
**Procedure:** Medical abortion.
**Clinical circumstances:** Longstanding diagnosis of schizophrenia with recurrent drug‐induced psychosis. Involuntary patient in mental health unit at the time of hearing.
**Social circumstances:** Single, no dependents, daughter interstate living with grandmother. No identification number, Centrelink income or phone number.
**Prior capacity assessments:** None mentioned.
**Evidence from:** *FXB*, psychiatrists, obstetrician and gynaecologists, mother.
**Nature of evidence:** *FXB* consistently told everyone that she wanted an abortion. *FXB* had previously been pregnant and had an uncomplicated labour and had given birth to a daughter. She had very limited involvement with her now 11‐year‐old daughter. Evidence from the psychiatrists noted that although her mental state had improved, she remained thought disordered, was difficult to engage, and had poor insight into mental illness. Her reasons for requesting an abortion were illogical and sometimes driven by delusional ideations. She was unable to demonstrate that she understood the risks and benefits of options, was overall unable to “… relay her reasoning in a consistently logical manner” and, therefore, lacked the capacity to consent [paragraph 42 of the judgment]. Evidence from the obstetrician and gynaecologist noted that *FXB* had “… full understanding of the fact that she is pregnant and the consequences of terminating her pregnancy” [paragraph 47 of the judgment]. She was believed to understand the process of abortion and appreciate the risks involved. She could consistently repeat back to the consultant her options and what was going to happen.
**Tribunal's reasoning for finding of capacity:** Accepted psychiatric evidence that her medication had brought her to baseline functioning and evidence of the obstetrician and gynaecologist that she understood the different procedures, risks and benefits.

### Capacity is decision‐specific

Capacity for decision making on abortion must be considered separately and distinctly from the capacity to decide other matters. Even though a woman may have impaired capacity and require a guardian for other treatment decisions, she may still have capacity to consent to a termination. In *BSE*, the tribunal noted that the Public Guardian had been making ongoing general health care decisions in its role as Statutory Health Attorney, and it also confirmed the appointment of a guardian to make decisions on accommodation and the provision of services. It noted that the termination decision was a “once off” decision which did not require ongoing planning as did decision making in those other areas.^17^ Similarly, in the case of *C*, it was noted that there was an ongoing appointment of a guardian for decision making on health care and services and an administrator for finances. Yet both women were found to have capacity to decide to have an abortion.

In *FXB*, the woman was an involuntary inpatient under the *Mental Health Act 2007* (NSW), which does not require a finding of incapacity for a patient to be treated involuntarily. It requires only that they have a mental illness or be mentally disordered so that their associated actions may result in harm to themselves or others.^18^ *FXB*'s symptoms warranted involuntary treatment, but she was still found to have capacity to consent to an abortion.

### Capacity test is functional, not based on diagnosis

A diagnosis of cognitive impairment does not necessitate a determination of incapacity. *BSE*, *AB* (in *C*) and *FXB* had all been diagnosed with mental illness, and *GKB* with an intellectual disability. *BSE*'s and *GKB*'s cognitive impairments meant that they were both receiving support from the National Disability Insurance Scheme for daily living. Nevertheless, in all four cases, a determination of capacity was made because the test for capacity is functional and not based on diagnosis. Thus, in every case examined, the women who had requested abortions had capacity to provide lawful consent.

### General understanding of the procedure

In applying the functional test of capacity, tribunals consider the clarity of the women's reasons for wanting a termination and whether they had a general (not technical or medical) understanding of the procedure. They also consider whether the women understood the nature of the options facing them. Potentially, the complexity or risk level of the particular procedure in any case could have an impact on the woman's understanding of it and, ultimately, the capacity determination.

Notably, no case examined concerned an abortion after 20 weeks, where the procedure may be more complex. Nor did we find any cases where an abortion was recommended for a woman for medical reasons but she refused. The capacity threshold to refuse an abortion, in the face of possible injury or death occurring, may be higher.^19^, ^20^

In *FXB*, since the tribunal had no power to consent to surgery for an involuntary patient under the *Mental Health Act 2007* (NSW), consent for a medical abortion was sought at 19 weeks. Although medical abortions are mostly performed up to nine weeks’ gestation, the guidelines from the Royal Australian and New Zealand College of Obstetricians and Gynaecologists recommend offering a choice of procedure up to 24 weeks’ gestation.^21^ However, performing this procedure for *FXB* at 19 weeks’ gestation was explained to be more complex. A medical abortion usually requires self‐administration of mifepristone and misoprostol 48 hours apart under medical supervision and with necessary follow‐up.^21^ In *FXB*'s case, hospitalisation and multiple doses of misoprostol were required. Despite this added complexity, *FXB* consistently repeated back to the consultant obstetrician and gynaecologist what was going to happen, and a finding of capacity was made.

### Differing medical opinions

In cases of uncertainty, it is important to consider evidence from a wide range of witnesses. In *FXB*, the opinions of the psychiatrists on capacity differed from those of the obstetrician and gynaecologist consultant and registrar. The psychiatrists believed that because of her schizophrenia, *FXB* could not relay her reasoning or weigh up the risks and benefits of the procedure in a “consistently logical manner”. However, the obstetrician and gynaecologist consultant and registrar responsible for the abortion, whose evidence the tribunal ultimately preferred, both found that *FXB* was consistent in her desire for a termination and understood the nature of the procedure, including the associated risks. *FXB*'s separate legal representative also submitted that, in all her discussions, *FXB* had demonstrated an understanding of the procedure and her other available options.

### Undue influence

In both *GKB* and *BSE*, questions were raised around undue influence, highlighting the need for vigilance around the issue of reproductive coercion.^22^ *GKB* had previously moved away from her family because of domestic violence, and it was reported that they may be disapproving of the pregnancy, albeit not yet accepting its reality. *BSE*'s guardian reported that, initially, she was concerned about her boyfriend seeking an abortion for her but that it was no longer an issue. In each case, the tribunal found that undue influence was not an operative factor in the decision to seek an abortion.

## Summary and conclusion

For adult women, an analysis of the limited number of recent cases reinforces that:

- capacity is always presumed;
- women can still have capacity to consent to an abortion, despite being found to lack capacity for other decisions; and
- a general understanding of the nature and effect of the procedure is sufficient to fulfil the functional test of capacity.

All of these cases are very much decided on their facts, and, as such, are not binding precedents for future tribunals and medical or legal practitioners. We further note that, due to the small number of cases, they cannot be said to be representative in any statistical sense. However, they can nevertheless be influential and provide a useful reference point in difficult cases. Although there may be scope for variation between jurisdictions, reliance in every jurisdiction on a functional capacity test suggests the tribunals’ reasoning and conclusions will likely align in cases concerning adult women. In the cases of women under the age of 18 years, different laws will apply.^23^, ^24^

When capacity is uncertain, medical practitioners should seek information and evidence from a variety of sources, including psychiatrists, other mental health practitioners, obstetricians and gynaecologists, Indigenous health workers, support workers, family and friends. Medical practitioners should be mindful of situations suggesting reproductive coercion and provide decision‐making support for women whose capacity has been questioned. In contentious cases, legal advice may be required.

## Open access

Open access publishing facilitated by Queensland University of Technology, as part of the Wiley – Queensland University of Technology agreement via the Council of Australian University Librarians.

## Competing interests

No relevant disclosures.

## Author contributions

Duffy JP: Conceptualization, investigation, formal analysis, supervision, writing – original draft, writing – review and editing. Boyle S: Conceptualization, formal analysis, writing – review and editing. Haining CM: Conceptualization, formal analysis, writing – review and editing.

## Provenance

Not commissioned; externally peer reviewed.

## References

[1] Health Act 1993 (ACT).

[2] Abortion Law Reform Act 2019 (NSW).

[3] Termination of Pregnancy Law Reform Act 2017 (NT).

[4] Termination of Pregnancy Act 2018 (Qld).

[5] Termination of Pregnancy Act 2021 (SA).

[6] Reproductive Health (Access to Terminations) Act 2013 (Tas).

[7] Abortion Law Reform Act 2008 (Vic).

[8] Public Health Act 2016 (WA).

[9] Abortion Legislation Reform Act 2023 (WA).

[10] Richards B. General principles of consent to medical treatment. In: White B , McDonald F , Willmott L , Then SN , editors. Health law in Australia, 4th ed. Sydney: Thomson Reuters, 2024; pp. 137‐164.

[11] Then SN , White B , Willmott L . Adults who lack capacity: substitute decision‐making. In: White B , McDonald F , Willmott L , Then SN ; editors. Health law in Australia, 4th ed. Sydney: Thomson Reuters, 2024; pp. 221‐286.

[12] Boyle S. Is the wisdom of a person's decision relevant to their capacity to make that decision? Monash University Law Review 2020; 46: 39‐57.

[13] Guardianship and Administration Act 1990 (WA) s 110ZD(1).

[14] Campbell M , Turudia, I . Capacity and consent under guardianship orders — not all medical treatment is equal. Australian Health Law Bulletin 2024; 32: 112‐115.

[15] Haining CM , Bowman‐Smart H , O’Rourke A , et al. The “institutional lottery”: institutional variation in the processes involved in accessing late abortion in Victoria, Australia. Women's Stud Int Forum 2023; 101: 102822. 39077555 10.1016/j.wsif.2023.102822 PMC11285594

[16] Then, S‐N , Duffy J , Bigby C , et al. Supported decision‐making: the current state of knowledge. Appendix A to: Bigby C , Carney T , Shih‐Ning T , et al. Diversity, dignity, equity and best practice: a framework for supported decision‐making. Canberra: Royal Commission into Violence, Abuse, Neglect and Exploitation of People with Disability, 2023. https://disability.royalcommission.gov.au/publications/diversity‐dignity‐equity‐and‐best‐practice‐framework‐supported‐decision‐making (viewed June 2025).

[17] BSE [2020] QCAT 494, at [21], [23], [25].

[18] Mental Health Act 2007 (NSW) ss 14‐15.

[19] Hunter and New England Area Health Service *v* A [2009] NSWSC 761.

[20] Application of a Local Health District; Re a Patient Fay [2016] NSWSC 624.

[21] Royal Australian and New Zealand College of Obstetricians and Gynaecologists . Clinical guideline for abortion care: an evidence‐based guideline on abortion care in Australia and Aotearoa New Zealand. Melbourne: RANZCOG, 2023. https://ranzcog.edu.au/wp‐content/uploads/Clinical‐Guideline‐Abortion‐Care.pdf (viewed May 2025).

[22] López Radrigán C. Disability and intersectionality: the construction of vulnerability in sexual and reproductive matters. In: Bach M , Espejo‐Yaksic N ; editors. Legal capacity, disability and human rights. Cambridge: Intersentia, 2023; pp. 50‐68.

[23] Secretary, Department of Health and Community Services *v* JWB and SMB (Marion's case) [1992] HCA 15, (1992) 175 CLR 218.

[24] Lim J , Peddibhotla R . The application of Gillick competency to abortion cases. Brisbane: Pro Bono Centre, University of Queensland; 2021. https://law.uq.edu.au/files/76128/REP_PBC_ChildrenbyChoice_GillickCompetency_20210901.pdf (viewed Nov 2024).

